# Ösophaguskarzinom: Prognoseverbesserung durch neoadjuvante Radiochemotherapie

**DOI:** 10.1007/s00066-021-01881-3

**Published:** 2021-11-25

**Authors:** Stefan Knippen, Marciana-Nona Duma

**Affiliations:** Klinik für Strahlentherapie und Radioonkologie, (Direktorin: Prof. Dr. med. A. Wittig), Universitätsklinikum Jena, Bachstr. 18, 07745 Jena, Deutschland

## Hintergrund

Die Rolle der neoadjuvanten Radiochemotherapie in der Behandlung des Ösophaguskarzinoms war lange Zeit Gegenstand kontroverser Diskussionen. Kritikpunkte vorheriger Studien betrafen v. a. das Studiendesign und oftmals auch eine geringe Fallzahl. Die Zusammenfassung der Ergebnisse verschiedener Studien in Metaanalysen lies einen Überlebensvorteil der mit RCT vorbehandelten Patienten vermuten, möglicherweise jedoch auf Kosten gesteigerter postoperativer Komplikationen. Die Veröffentlichung der Ergebnisse der randomisierten Phase-3-CROSS-Studie aus den Jahren 2012 und 2015 war und ist noch immer in der Behandlung des lokal fortgeschrittenen Ösophaguskarzinoms als „practice-changing“ anzusehen. Eyck et al. untermauern dies in der aktuellen Veröffentlichung mit den nun vorliegenden 10-Jahres-Daten.

## Patienten und Methodik

366 Patienten wurden zwischen 2004 und 2008 randomisiert mit einer neoadjuvanten RCT (nRCT; GD 41,4 Gy, ED 1,8 Gy; 5 wöchentliche Kurse Carboplatin AUC2/Paclitaxel 50 mg/m^2^) vorbehandelt und anschließend reseziert oder primär operiert (S). Die Strahlentherapie wurde als 3‑D-konformale Therapie mit einem kraniokaudalen Sicherheitssaum von 4 cm und einem radialen Saum von 1,5 cm durchgeführt. Es wurden sowohl Patienten mit Plattenepithelkarzinomen (23 %) als auch mit Adenokarzinomen (75 %) des Tumorstadiums cT1 cN1 cM0 oder cT2‑3 cN0–1 cM0 an 8 niederländischen Institutionen behandelt, wobei ein Großteil der Fälle als cT3 (84 % nRCT; 78 % S) und cN1 (65 % bzw. 64 %) eingestuft wurde. Die Lokalisation der Tumoren lag in einem Großteil der Fälle im distalen Ösophagus (58 % nRCT, 57 % S) und im ösophagogastralen Übergang (22 % nRCT, 26 % S). Insgesamt sind beide Studienarme als gut balanciert zu bezeichnen. Die Resektion erfolgte 4–6 Wochen nach der neoadjuvanten Vorbehandlung bzw. unmittelbar nach der Randomisierung. Die Patienten wurden im ersten Jahr alle 3 Monate, im zweiten Jahr alle 6 Monate und anschließend bis zum fünften Jahr jährlich nachgesorgt. Nach Abschluss des fünften Jahrs erfolgten Patientenvorstellungen bei Symptomen. Zur Erhebung der 10-Jahres-Daten wurden die betreuenden Hausärzte kontaktiert. Die Auswertung wurde folgerichtig als Intention-to-treat-Analyse durchgeführt. Primärer Endpunkt war das Gesamtüberleben (OS), sekundäre Endpunkte die krankheitsspezifische Letalität sowie die kumulativen Inzidenzen lokoregionärer Rezidive und Fernmetastasen. Die Berechnung des Follow-ups erfolgte nach reversem Kaplan-Meier-Verfahren, das Gesamtüberleben nach Kaplan-Meier, Vergleiche erfolgten nach log-Rang. Weiterhin erfolgte die uni- und multivariate Analyse von vordefinierten Subgruppen nach dem Cox-proportional-hazards-Modell.

## Ergebnisse

Mit Ausnahme eines Patienten liegt für alle Behandelten ein minimaler Nachbeobachtungszeitraum von 120 Monaten vor. Die Vorbehandlung mit der nRCT führt zu einer signifikanten Verbesserung des Gesamtüberlebens (HR 0,7, *p* = 0,004). So leben im nRCT-Arm nach 10 Jahren noch 38 % der Behandelten, im Arm der allein resezierten Patienten noch lediglich 25 %. Noch deutlicher wird der Überlebensvorteil, wenn man die Unteranalyse nach Histologie betrachtet: Nach nRCT leben nach 10 Jahren noch 46 % der Patienten mit plattenepithelialen Karzinomen, nach alleiniger Op. jedoch nur 23 %. Beim Adenokarzinom verbessert die Vorbehandlung das Überleben um absolut 10 % (nRCT 36 % vs. S 26 %). Die Auswertung der krankheitsspezifischen Letalität zeigt, dass der Überlebensvorteil durch eine Reduktion der Wahrscheinlichkeit, am Ösophaguskarzinom zu versterben, bedingt ist (HR 0,6, Risiko nach 10 Jahren 47 % nRCT bzw. 64 % S). Die Effekte scheinen durch die verbesserte lokale Kontrolle bedingt zu sein; es entwickelten nach nRCT nur 8 % ein isoliertes lokoregionäres Rezidiv bzw. 13 % ein lokoregionäres Rezidiv bei gleichzeitiger Fernmetastasierung. In der Gruppe der allein operierten Patienten waren dies 18 % bzw. 22 %. In beiden Gruppen traten etwa 85 % der Rezidive innerhalb der ersten 3 Jahre auf, keines aber nach dem sechsten Jahr mehr. Die Rate an alleinigen Fernmetastasen wurde durch die Therapie nicht beeinflusst (nRCT 27 %, S 28 %). Die zeitliche Kartierung des Hazard Ratio für OS zeigt, dass der Effekt der nRCT sich innerhalb der ersten 5 Jahre auswirkt, danach ist das HR ≥ 1.

## Schlussfolgerung der Autoren

Die neoadjuvante RCT des operablen, lokal fortgeschrittenen Ösophaguskarzinoms oder des Karzinoms des ösophagogastralen Übergangs nach dem Konzept der CROSS-Studie führt zu einer Verbesserung des OS ohne erhöhte therapiebedingte Sterblichkeit. Der Therapieeffekt persistiert auch über 10 Jahre nach einer solchen Therapie.

## Kommentar

Ösophaguskarzinome gehören unter den gastrointestinalen Neubildungen zu denjenigen mit der schlechtesten Prognose. Das 5‑Jahres-Gesamtüberleben beträgt, weltweit betrachtet, lediglich 15–25 %. Bei Männern ist das Ösophaguskarzinom überhaupt die sechsthäufigste krebsbedingte Todesursache [1]. Die zwei häufigsten histologischen Subtypen, Plattenepithelkarzinome und adenosquamöse Karzinome, variieren in ihrer Häufigkeit je nach geografischer Region, wobei in unseren Breitengraden und in Nordamerika die Adenokarzinome überwiegen [2]. Die unbefriedigenden Therapieergebnisse in der Behandlung lokal fortgeschrittener Ösophaguskarzinome haben zahlreiche Studien zu neoadjuvanten und adjuvanten Therapieregimen hervorgebracht, denn ohne Vorbehandlung sind trotz adäquatem Staging R1-Resektions-Raten in mehr als 25 % der Fälle zu erwarten [3].

Global betrachtet unterscheidet sich die Art des neoadjuvanten Vorgehens, begründet durch die unterschiedliche Prävalenz der histologischen Subtypen. So gibt man im asiatischen Raum mit hoher Prävalenz von Plattenepithelkarzinomen den Chemotherapien den Vorzug. Das JCOG9204-Protokoll wies hierbei die Wirksamkeit einer postoperativen Cisplatin/5-FU-Kombination nach [4], JCOG9907, publiziert 2012, die Verbesserung des Überlebens durch die neoadjuvant applizierte Chemotherapie [5], die seither im Japan den Standard darstellt. In Europa und Amerika etablierte die erste Veröffentlichung der CROSS-Studie im selben Jahr die neoadjuvante Radiochemotherapie (RCT), die ihre Wirksamkeit bei beiden histologischen Entitäten unter Beweis stellte, mit höherer Wirksamkeit jedoch bei den Plattenepithelkarzinomen. Beeindruckend waren die deutliche Verbesserung der R0-Resektionsrate (92 % vs. 69 %), die Rate an pathologischen Komplettremissionen im nRCT-Arm von 29 %, mit einer pCR-Rate von 49 % bei den plattenepithelialen Karzinomen, sowie die durch die nRCT verringerte Rate an postoperativ verbliebenen Lymphknotenmetastasen. Schon nach 45 Monaten Nachbeobachtung zeigte sich eine deutliche Verbesserung des Gesamtüberlebens als auch, dass die Mehrheit der verstorbenen Patienten an ihrer lokal rezidivierten Erkrankung verstarb [6]. Aktuell kann Patienten mit pathohistologisch nachgewiesener Resterkrankung (ypT1 oder ypN1) nach der nRCT die adjuvante Immuncheckpointblockade mit Nivolumab angeboten werden, die das mediane krankheitsfreie Überleben von 11 auf 22 Monate verdoppeln kann [7] und so zu einer weiteren Prognoseverbesserung beiträgt.

Ein Kritikpunkt, dem sich die CROSS-Studie trotz der hervorragenden 10-Jahres-Daten stellen muss, ist, dass die therapiebedingten Nebenwirkungen nur in den ersten 2 Jahren dokumentiert wurden. Denn Nebenwirkungen, vor allem am kardiopulmonalen System, manifestieren sich durchaus über diesen Beobachtungszeitraum hinaus. Und ein Teil von ihnen wird bereits nach 2–3 Jahren evident [8]. Der auch nach 10 Jahren nachweisbare Überlebenseffekt der nRCT manifestiert sich vor allem in den ersten 5 Jahren, sodass eine durch die RT bedingte kardiale Letalität in größerem Maße unwahrscheinlich ist. Dies wird auch durch die Literatur bestätigt [9]. Des Weiteren ergibt sich durch die heutzutage regelhaft angewendeten IMRT-Techniken eine „En-passant“-Schonung von Herz und Lungen [10].

Die Ergebnisse der ersten CROSS-Publikation haben die Behandlungsabfolge beim lokal fortgeschrittenen Ösophaguskarzinom nachhaltig verändert. Ein Streitpunkt blieben jedoch oft Karzinome des ösophagogastralen Übergangs, basierend auf den 6 Jahre zuvor publizierten Daten des MAGIC-Trials, der neben Magenkarzinomen auch 25 % Karzinome des distalen Ösophagus eingeschlossen hatte und durch eine perioperative ECF-Chemotherapie das Gesamt- und das progressionsfreie Überleben verbesserte [11]. Schon 3 Jahre später legten jedoch Daten der Essener Studie nahe, dass die nRCT bei EGJ-Tumoren der alleinigen neoadjuvanten Chemotherapie vorzuziehen sei. Aufgrund der langsamen Rekrutierung erreichte jedoch das vorzeitig geschlossene Protokoll keine statistische Signifikanz [12].

Die neoadjuvante Chemotherapie wird trotzdem in dieser Frage oft in Stellung gebracht und weiterhin wissenschaftlich untersucht, zuletzt in der randomisierten Phase-II/III-FLOT4-Studie. Diese zeigte dabei, dass das FLOT-Protokoll dem ECF-Schema vorzuziehen ist [13]. Eine durchaus bestehende antitumorale Wirksamkeit der Chemotherapie wird jedoch im Vergleich zur nRCT mit einer deutlich höheren Toxizität erkauft. Des Weiteren müssen sich die Langzeitergebnisse des FLOT-Protokolls noch beweisen. Rational betrachtet gibt es angesichts der vorliegenden 10-Jahres-Daten eigentlich keinen Grund, die alleinige Chemotherapie in der Behandlung der EGJ-Karzinome einzusetzen.

## Fazit

Die 2012, 2015 und nun mit aktuellen 10-Jahres-Daten veröffentlichten Ergebnisse der CROSS-Studie haben die neoadjuvante RCT beim Ösophaguskarzinom fest etabliert. Bei moderaten, therapieassoziierten Nebenwirkungen erreicht die Therapie nach dem CROSS-Schema eine deutlich verbesserte Prognose, ohne die Sterberate zu erhöhen. Der Effekt des verbesserten OS resultiert vor allem aus einer verbesserten lokoregionären Kontrolle innerhalb der ersten 5 Jahre. Der hier erreichte Überlebensvorteil bleibt mindestens 10 Jahre bestehen. CROSS ist das einzige randomisierte Protokoll für diese Erkrankungsentitäten mit einer Nachbeobachtungszeit von > 10 Jahren. Die auf der Basis des CROSS-Protokolls erreichbaren Ergebnisse sollten allen Betroffenen im Sinne des „shared decision-making“ empfohlen werden.

*Stefan Knippen und Marciana-Nona Duma, Jena*
